# *Lactobacillus rhamnosus* GG powder supplementation alleviates intestinal injury in piglets challenged by porcine epidemic diarrhea virus

**DOI:** 10.3389/fcimb.2024.1371916

**Published:** 2024-04-23

**Authors:** Zhaoyang Xu, Qian Zhang, Mengjun Wu, Yanyan Zhang, Zhonghua Li, Hanxiao Li, Chenmin Yu, Xiaohan Zhang, Di Zhao, Lei Wang, Yongqing Hou, Tao Wu

**Affiliations:** Hubei Key Laboratory of Animal Nutrition and Feed Science, Wuhan Polytechnic University, Wuhan, China

**Keywords:** *Lactobacillus rhamnosus* GG, PEDV, gut health, gut functions, piglets

## Abstract

Porcine epidemic diarrhea virus (PEDV) has become a challenging problem in pig industry worldwide, causing significant profit losses. *Lactobacillus rhamnosus* GG (LGG) has been regarded as a safe probiotic strain and has been shown to exert protective effects on the intestinal dysfunction caused by PEDV. This study evaluated the effect of LGG on the gut health of lactating piglets challenged with PEDV. Fifteen piglets at 7 days of age were equally assigned into 3 groups (5 piglets per group): 1) control group (basal diet); 2) PEDV group: (basal diet + PEDV challenged); 3) LGG + PEDV group (basal diet + 3×10^9^ CFU/pig/day LGG + PEDV). The trial lasted 11 days including 3 days of adaptation. The treatment with LGG was from D4 to D10. PEDV challenge was carried out on D8. PEDV infection disrupted the cell structure, undermined the integrity of the intestinal tract, and induced oxidative stress, and intestinal damage of piglets. Supplementation of LGG improved intestinal morphology, enhanced intestinal antioxidant capacity, and alleviated jejunal mucosal inflammation and lipid metabolism disorders in PEDV-infected piglets, which may be regulated by LGG by altering the expression of TNF signaling pathway, PPAR signaling pathway, and fat digestion and absorption pathway.

## 1 Introduction

Porcine epidemic diarrhea (PED) is a highly contagious intestinal disease caused by the porcine epidemic diarrhea virus (PEDV) and is considered one of the most significant threats to the pig farming industry. In recent years, the global spreading of PED has caused enormous economic losses to the pig farming industry. PEDV, as well as transmissible gastroenteritis virus and porcine enteric A coronavirus, belongs to the genus α-coronavirus in the family Coronaviridae (Gong et al., 2017). The main PEDV transmission route is fecal-oral, but airborne transmission via the fecal-nasal route may play a role in pig-to-pig and farm-to-farm spread (Gerber et al., 2014; Yuan et al., 2022). Piglets up to 7 days of age are particularly susceptible to the virus and thus cause acute diarrhea, vomiting, dehydration and high mortality in newborn piglets (Sun et al., 2016). In recent years, various mutations and recombinations have occurred between different strains and even between PEDV and other coronaviruses. The lack of updated effective vaccination in controlling PEDV infections has caused continuous problems in the pig industry (Du et al., 2019).

*Lactobacillus rhamnosus* GG (LGG), a Gram-positive parthenogenetic anaerobic bacterium first extracted from the gastrointestinal tract of healthy human beings in 1983 (Shi et al., 2019), is one of the most widely studied probiotic organisms for atopic diseases. The main characteristics of LGG are the strong ability to survive and reproduce under low pH conditions of gastric acid and bile, easy to colonize and function in the intestinal tract, with a high level of immunological activity (Hou et al., 2023). Some studies have shown that colonization of LGG in neonatal mice enhances intestinal functional maturation and IgA production and confers lifelong health consequences on protection from intestinal injury and inflammation (Yan et al., 2016). Oral administration of *Lactobacillus rhamnosus* powder to mice alleviated antibiotic-induced intestinal injury (Cresci et al., 2013). Moreover, LGG may be effective in ameliorating E. coli K88-induced diarrhea in weaned piglets by modulating the intestinal flora, enhancing the intestinal antibody defenses, and modulating the production of systemic inflammatory factors (Zhang et al., 2010). In addition, dietary *Lactobacillus rhamnosus* GG supplementation improves the mucosal barrier function in the intestine of weaned piglets challenged by Porcine Rotavirus (Mao et al., 2016). Moreover, studies have shown that treatment with *L. rhamnosus* can significantly increase the villus height of ileum, promote the proliferation of T Cell proliferation, and therefore improve the absorption function and immune capacity of intestines in piglets (Shonyela et al., 2020).

*Lactobacillus rhamnosus* has been applied in medical research and animal husbandry production, but there are few reports on the resistance of *Lactobacillus rhamnosus* powder to PEDV infection. Our research group has previously established the intestinal injury model of PEDV-infected piglets. Therefore, the present study aimed to explore the protective effect of *Lactobacillus rhamnosus* GG powder on the intestinal tract of PEDV-infected piglets.

## 2 Materials and methods

### 2.1 Animal care and diets

All animal procedures used in the present study were approved by the Institutional Animal Care and Use Committee of Wuhan Polytechnic University (Index number: 202205003). A total of 15 healthy 7-day-old piglets (Duroc × Landrace × Yorkshire) were used for the experiment. Piglets were housed separately in two environment-controlled nursery rooms (28-30°C) to avoid cross-infection and given ad libitum access to water throughout the study. The freeze-dried *Lactobacillus rhamnosus* GG powder containing 3 ×10^9^ colony forming units (CFU)/g was purchased from Hubei Haohua biotechnology Co., Ltd (Wuhan, China), and stored in the sealed packet at 4°C until used. A commercial milk replacer from Shanghai Nouriz Dairy Co., Ltd (Shanghai, China) was served as a basal diet for piglets, formulated to satisfy the requirements of suckling piglets. Before feeding, the milk replacer was dissolved in warm water (45–55°C) to form a liquid feed (dry matter content of 20%). Pigs were fed with the liquid feed every 3 h between 8:00 am and 8:00 pm.

### 2.2 Experiment design

The healthy piglets (half male and half female) at 7 day of age (Duroc × Landrace × Yorkshire, BW = 3.51 ± 0.33 kg) were used in this experiment. Piglets were housed in clean pens with strict control of cross-infection.The trial lasted 11 days (D0-D10), including three days of adaptation (D1-D3). The treatment with LGG was from D4 to D10. The piglets were orally administered with PEDV at a dose of 10^6^ TCID_50_ (50% tissue culture infectious dose) per pig on D8, We used the dose of infection in our previous study and pretest (Zhang et al., 2022; Song et al., 2024), while the control group was orally administered with an equal volume of PBS solution. Fifteen healthy crossbred piglets were chosen and the commercially available milk replacer (Nouriz shanghai, China) was used as a basic diet. After 3-day adaptation, the piglets were assigned randomly, based on body weight (BW) and litter origin, to 3 groups: (5 piglets/group): 1) control group - piglets fed the basal diet; 2) PEDV group: piglets fed the basal diet, and were orally administered with 3 ml 1×10^6^ TCID_50_/pig PEDV; 3) LGG+PEDV group-piglets fed the basal diet supplemented with 50 mg/kg BW LGG supplementation in diet, and were orally administered with 3 ml 1×10^6^ TCID_50_/pig PEDV.

### 2.3 Samples collection

**Figure 1:**
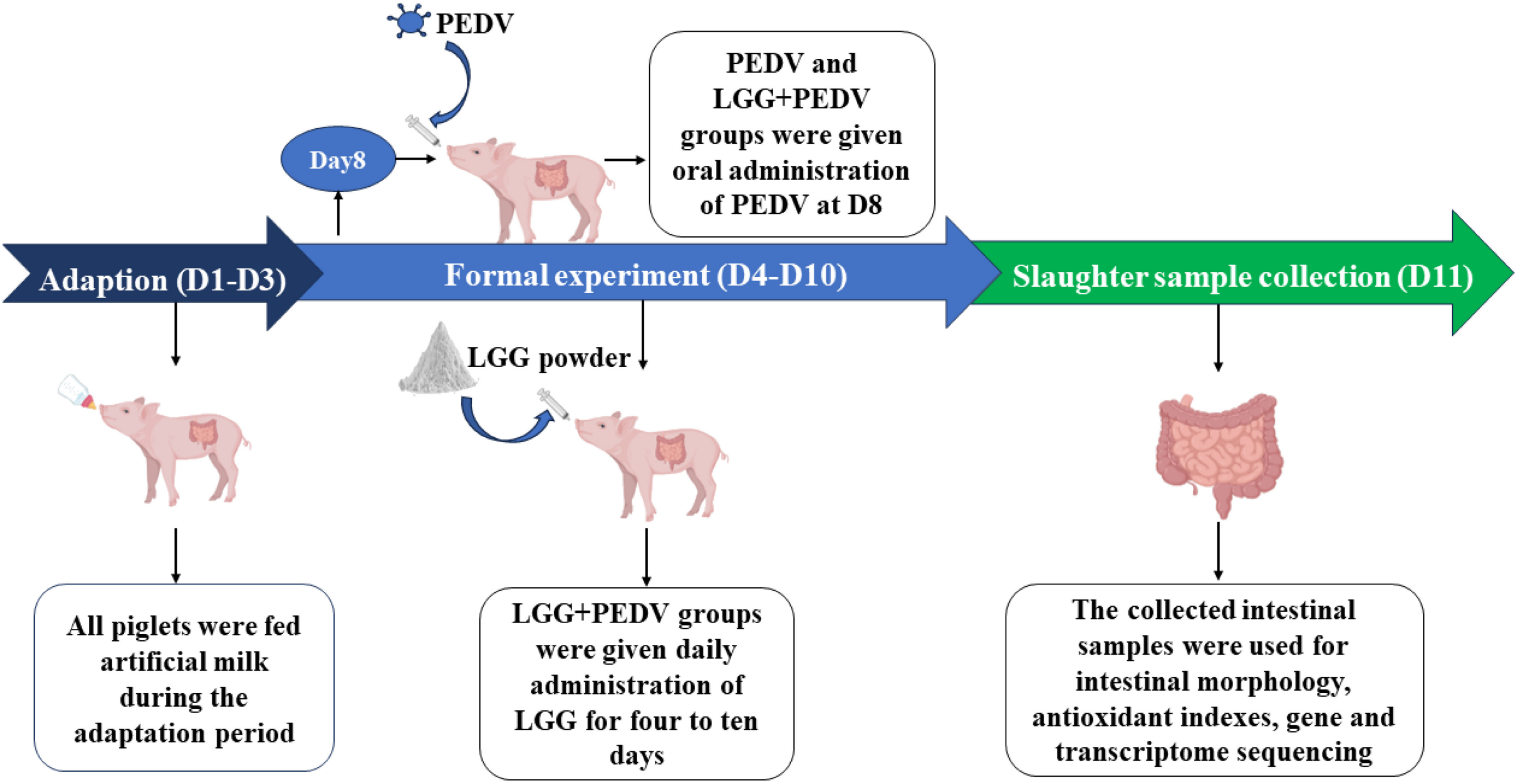
Workflow for PEDV challenge, administration of LGG powder, and sample collection in piglets.

Intestine sample was collected from the jugular vein of piglets on D11 and then all animals were slaughtered under sodium pentobarbital anesthesia (50 mg/kg BW, iv). The abdomen of piglets was opened immediately and the small intestine was separated from the mesentery onto a chilled stainless-steel tray, then the 5-cm segment samples were cut respectively at distal duodenum, mid-jejunum and mid-ileum. The samples were gently flushed with ice-cold PBS and placed in chilled formalin solution (10%), mucosa was collected by scraping using a sterile glass microscope slide at 4°C, rapidly frozen in liquid nitrogen, then processed by embedding and staining for the observation of intestinal morphology. All the samples were collected within 10 min. Intestinal tissues were stored at −80°C until analysis. Piglet challenge PEDV, administration of LGG powder and sample collection process are shown in **Figure 1**.

### 2.4 Intestinal morphology

The procedure of preparing samples for measuring intestinal morphology was performed as described previously (Wu et al., 2018). Briefly, the samples were dehydrated and embedded in paraffin, sectioned at a thickness of 4 mm, and stained with haematoxylin and eosin. Morphological examination was conducted with a light microscope (Leica microsystems, Wetzlar, Germany) with the Leica Application Suite image analysis software (Leica microsystems, Wetzlar, Germany). Eight areas per slide were inspected randomly in a double-blind manner. Intestinal villus height and crypt depth were measured to calculate the ratio of villus height to crypt depth.

### 2.5 Antioxidant capacity of intestine mucosa

Mucosa of duodenum, jejunum, ileum, and colon were used for analysis of antioxidative enzymes and oxidation-related products. The activities of catalase (CAT), total superoxide dismutase (T-SOD), myeloperoxidase (MPO), and the concentration of hydrogen peroxide (H_2_O_2_) and malondialdehyde (MDA) were determined by using commercially available kits (Nanjing Jiancheng Bioengineering Institute, Nanjing, China), according to the protocols of manufacturer.

### 2.6 Quantitative RT-PCR assay

The gene expression levels were quantitated using the method of qRT-PCR assays and the primers used in the present study was shown in **Table 1**, which were either verified experimentally using the conditions in previous publications including the housekeeping gene encoding ribosomal protein L19 (RPL19) or were designed using Primer Express software version 3.0 (Applied Biosystems). The qRT-PCR was performed using the SYBR ^®^ Premix Ex Taq TM (Takara, Dalian, China) on an Applied Biosystems 7500 Fast qRT-PCR System (Foster City, CA). The total volume of PCR reaction system was 50 μL, containing 0.2 µM of each primer, 25 µL of SYBR ^®^ Premix Ex Taq TM (2×), and 4 µL of cDNA. All PCR assays were performed in triplicate on a 96-well real-time PCR plate (Applied Biosystems) under the following conditions (two-step amplification): 95°C for 30 sec, followed by 40 cycles of 95°C for 5 sec and 55-65°C for 31 sec depending on the melting temperature of each pair of primers. A subsequent melting curve (95°C for 15 sec, 60°C for 1 min and 95°C for 15 sec) with continuous fluorescence measurement was constructed. Data were analyzed using the 2-ΔΔCt method as described (Yi et al., 2016).

**Table 1:** Primer sequences of qRT-PCR.

Gene	Sequences (5′–3′)	Product (bp)	Accession Numbers	References
Viral Gene				
* PEDV M*	F: TCCCGTTGATGAGGTGAT	231	KT021228	Present study
	R: AGGATGCTGAAAGCGAAAA			
* PEDV N*	F: TTGGTGGTAATGTGGCTGTTC	180	KT021228	Present study
	R: TGGTTTCACGCTTGTTCTTCTT			
Anti Inflammatory				
* RPL19*	F: AACTCCCGTCAGCAGATCC	147	XM_003131509.5	Present study
	R: AGTACCCTTCCGCTTACCG			
* MX1*	F: AGTGCGGCTGTTTACCAAG	150	NM_214061.2	(Li et al., 2023)
	R: TTCACAAACCCTGGCAACTC			
* ISG15*	F: AGCATGGTCCTGTTGATGGTG	164	NM_001128469.3	(Song et al., 2024)
	R: CAGAAATGGTCAGCTTGCACG			
* OASL*	F: GGCACCCCTGTTTTCCTCT	139	NM_001031790.1	(Li et al., 2023)
	R: AGCACCGCTTTTGGATGG			
* IRF7*	F: CAGAAGCAGCTCCACTACAC	120	XM_021076252.1	Present study
	R: CTCCCAGTAGACTTTGCACTT			
* IRF3*	F: CCTACACTCCTGGGAAGGATAA	135	NM_213770.1	Present study
	R: CATAGATCTTGTGTGGGTCGTG			
* IL-8*	F: TTCGATGCCAGTGCATAAATA	176	NM_213867.1	(Zhang et al., 2023)
	R: CTGTACAACCTTCTGCACCCA			
* IL-1β*	F: CAACGTGCAGTCTATGGAGT	372	XM_021085847.1	(Li et al., 2023)
	R: GAGGTGCTGATGTACCAGTTG			
* CXCL2*	F: CGGAAGTCATAGCCACTCTCAA	143	NM_001001861.2	(Song et al., 2024)
	R: CAGTAGCCAGTAAGTTTCCTCCATC			
Lipid Metabolism				
* ACADL*	F: GGATGGAAGTGACTGGATTCTC	107	NM_213897.1	Present study
	R: GAGAGCGAGCTTCACGATTT			
* ACOX1*	F: CCTATGACGGGAATGTGTATG	101	XM_021066020.1	Present study
	R: GACTTCAGGTGCTTGTGGTAAG			
* APOA1*	F: CCTTGGCTGTGCTCTTCCTC	100	NM_214398.1	(Zhang et al., 2019)
	R: ACGGTGGCAAAATCCTTCAC			
* APOC3*	F: CTAACCAGCGTGAAGGAGTC	116	XM_021102248.1	Present study
	R: CAGAAGTCGGTGAACTTGCC			
* EHHADH*	F: CGGGATGAGATCCTTGACCG	286	XM_021069979.1	Present study
	R: CTTTCAGGGGAGGGTTTCCC			
* FABP1*	F: AACTTCTCCGGCAAATACCAA	129	NM_001004046.2	Present study
	R: ATTCTGCACGATTTCCGATG			
* FABP2*	F: CCTTGGCTGTGCTCTTCCTC	139	NM_001031780.1	Present study
	R: ACGGTGGCAAAATCCTTCAC			
* ACSL3*	F: CTAACCAGCGTGAAGGAGTC	150	XM_021074569.1	(Zhang et al., 2019)
	R: GTATCCACCTTCTTCCCAGTTCTTT			
* SLC27A2*	F: TTTTCAGCCAGCCACTTTTG	109	NM_001278777.1	(Zhang et al., 2019)
	R: CATTTGGTTTCTGGGGAGAGTT			
* APOB*	F: GGGATGATGGCACAGGTTACA	123	NM_001375388.1	Present study
	R: TGACGTGGACTTGGTGCTTT			
* ADHFE1*	F: GTGGAACCCACCCAGAGAAG	221	XM_003125603.4	Present study
	R: GGGACTGCAATCAGAGGCTT			
*PPARGC1A*	F: GATCACGTTCAAGATCTCCCTAC	116	XM_021100444.1	Present study
	R: AGACTCCCGCTTCTCATACT			
* CXCR5*	F: GAGAACCAAGCGGAGACCAA	108	XM_003129915.4	Present study
	R: CACCACCCCTACGTAACACC			
* CCL4*	F: ATGAAGCTCTGCGTGACTGT	147	NM_213779.1	Present study
	R: AGTCACGAAGTTGCGAGGAA			
* PPARG*	F: AGGACTACCAAAGTGCCATCAAA	142	XM_005669788.3	Present study
	R: GAGGCTTTATCCCCACAGACAC			

### 2.7 RNA sequencing analysis

Total RNA was isolated from the Jejunum tissue using TRIzol reagent (Invitrogen, Carlsbad, CA, USA) following the manufacturer’s instructions. The integrity and purity of the total RNA was checked before construction of the sequencing library. The NEBNext Ultra RNA Library Prep Kit for Illumina (New England Biolabs, Ipswich, MA, USA) was used to generate the sequencing libraries, which were sequenced on an Illumina Hiseq platform (Illumina, San Diego, CA, USA) to generate paired-end reads. Thereafter, we used DESeq2 R package (1.18.1) to profile gene expression in the PEDV vs. Contral and LGG+PEDV vs. PEDV. Differential expression genes (DEGs) were filtered based on a *P*-value < 0.05 and a fold-change ≥1.5 or ≤ 0.67. Kyoto Encyclopedia of Genes and Genomes (KEGG) pathway analysis was carried out by Python Scipy based on differentially expressed genes, and the first 20 pathways with the smallest *P*- adjust, the most significant enrichment, were selected for display. A *P*-value < 0.05 was considered as a significance threshold.

### 2.8 Statistical analysis

All data were analyzed by one-way ANOVA using the GLM procedure of SPSS 20.0 software appropriate for a 2×2 factorial design (SPSS Inc. Chicago, IL, USA). The factors of models included the main effects of LGG treatment (supplemented or unsupplemented with LGG in the diet) and the PEDV challenge. *P*<0.05 was considered to indicate statistical significance, and *P*<0.10 was considered to indicate statistical tendency. All data were expressed as mean ± standard deviation.

## 3 Results

### 3.1 Intestinal morphology

**Figure 2:**
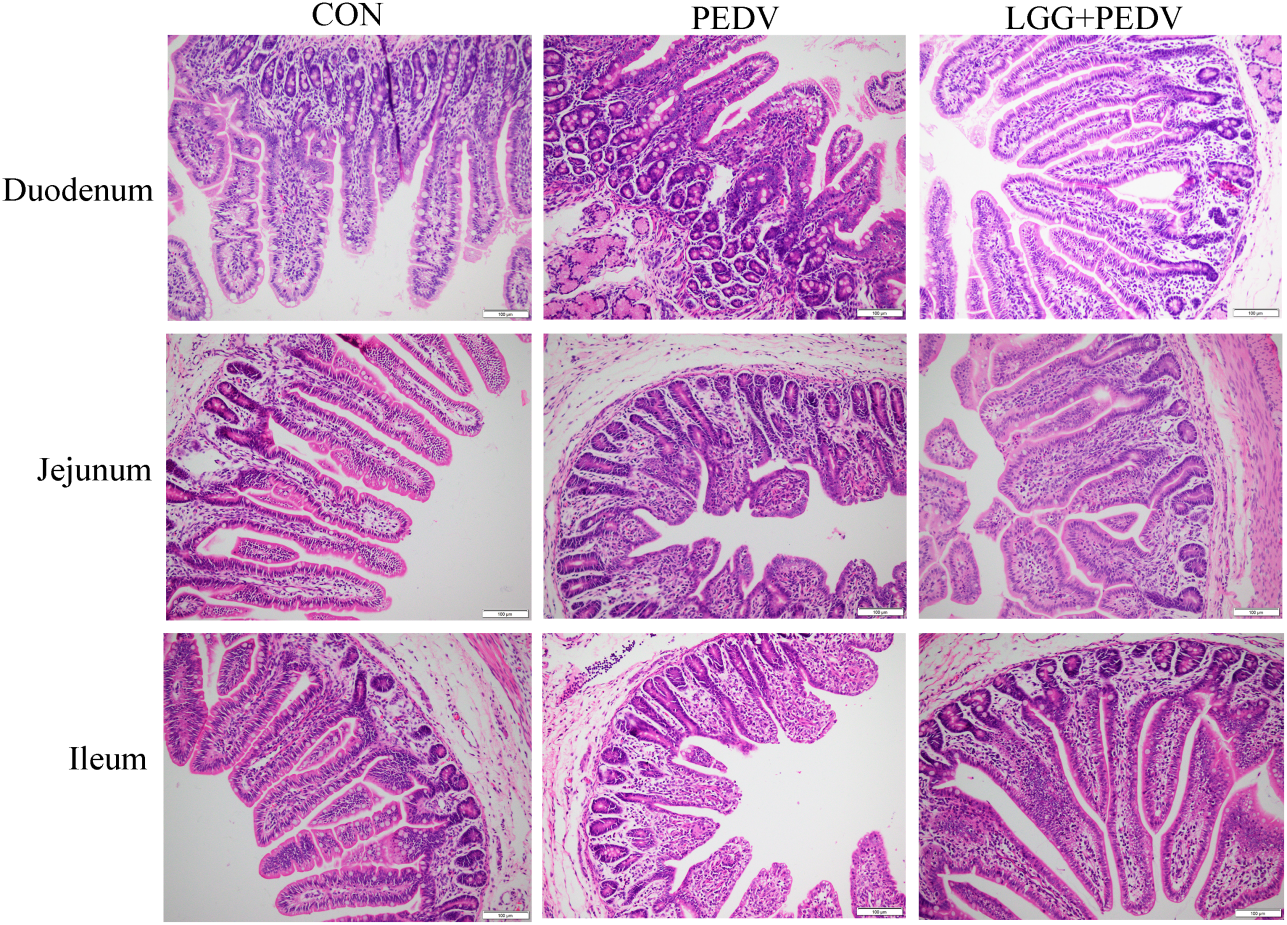
Effects of *Lactobacillus rhamnosus* GG on the intestinal morphology of piglets.

Small intestine is the main place for animals to digest and absorb nutrients. The structure and morphology of intestinal mucosa are closely related to the intestinal health of animals. In order to investigate the effect of LGG powder on intestinal morphology of PEDV-infected piglets, we observed and analyzed the villus growth in the small intestine of piglets. The microscopic pictures of intestinal mucosal are shown in **Figure 2**. The effects of LGG on the intestinal morphology of piglets is shown in **Table 2**. Compared with none-challenged piglets, PEDV infection decreased villus height/crypt depth ratio in all small intestinal segments (*P*< 0.05), decreased the villus height, and increased the crypt depth in the jejunum and ileum (*P*< 0.05). LGG supplementation decreased crypt depth and increased villus height/crypt depth ratio in the jejunum and ileum (*P*< 0.05), and increased the villus height in the ileum, as compared to the PEDV group.

**Table 2:** Effects of *Lactobacillus rhamnosus* GG on the intestinal morphology of piglets.

Item	Control	PEDV	LGG+PEDV	*P-value*
Duodenum				
Villus height (μm)	441.47 ± 73.00	367.02 ± 43.41	368.49 ± 61.94	0.130
Crypt depth (μm)	115.43 ± 16.34	120.54 ± 9.43	119.32 ± 5.69	0.760
VH/CD	3.94 ± 0.70^a^	3.04 ± 0.21^b^	2.98 ± 0.36^b^	0.010
Jejunum				
Villus height (μm)	436.95 ± 61.84^a^	279.44 ± 44.43^b^	257.31 ± 54.80^b^	<0.01
Crypt depth (μm)	110.66 ± 14.94^b^	149.61 ± 22.72^a^	104.20 ± 13.90^b^	<0.01
VH/CD	4.06 ± 0.43^a^	1.95 ± 0.66^c^	2.47 ± 0.27^b^	<0.01
Ileum				
Villus height (μm)	391.74 ± 25.68^a^	212.75 ± 17.33^c^	282.56 ± 56.92^b^	<0.01
Crypt depth (μm)	109.96 ± 5.68^b^	128.41 ± 9.99^a^	114.31 ± 8.08^b^	<0.01
VH/CD	3.69 ± 0.20^a^	1.74 ± 0.22^c^	2.77 ± 0.73^b^	<0.01

VH, villus height; CD, crypt depth; Values within a row with different letters differ significantly (P < 0.05).

### 3.2 Intestinal antioxidant capacity

**Figure 3:**
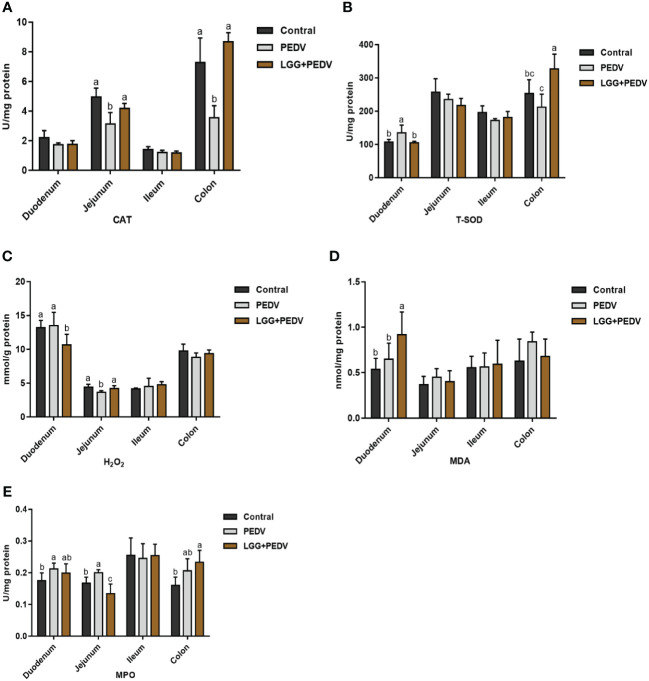
Effects of *Lactobacillus rhamnosus* GG on the intestinal antioxidant capacity of piglets **(A)** CAT, catalase; **(B)** T-SOD, total superoxide, and dismutase; **(C)** H_2_O_2_, hydrogen peroxide; **(D)** MDA, malondialdehyde. **(E)** MPO, Myeloperoxidase. ^a,b,c,^ Values within a column not sharing a common superscript letter indicate significant difference at *P* < 0.05.

Several representative antioxidant enzymes and oxidative damage products were detected in this experiment to reveal the effect of PEDV and LGG on intestinal antioxidant capacity in the intestinal tract of piglets, as shown in **Figure 3**. Compared with none-challenged piglets, the PEDV infection significantly increased MPO activity in the duodenum (*P*<0.05). The activity of CAT in the jejunum was significantly decreased in PEDV group (*P*<0.05). PEDV infection significantly decreased CAT activity in the colon (*P*< 0.05). Compared with the PEDV group, the LGG supplementation decreased MPO activity, in the duodenum. Compared with the PEDV group, the LGG supplementation significantly decreased the H_2_O_2_ concentration in the duodenum, increased CAT activity in the jejunum (*P*< 0.05). PEDV infection and LGG supplementation has no significantly effect in the ileum. LGG supplementation could significantly increased CAT and T-SOD activity in the colon (*P*<0.05).

### 3.3 Regulation of gene expression

**Figure 4:**
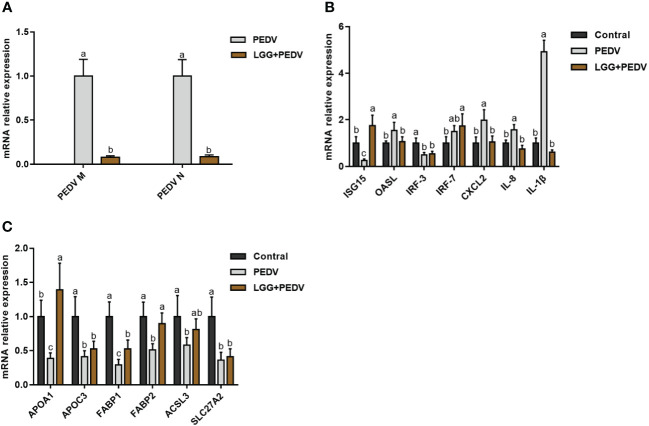
**(A)** Effects of *Lactobacillus rhamnosus* GG on the viral gene expression in the jejunum of piglets. **(B)** Effects of *Lactobacillus rhamnosus* GG on the Anti-Inflammatory gene expression in the jejunum of piglets. **(C)** Effects of *Lactobacillus rhamnosus* GG on the lipid metabolism gene expression in the jejunum of piglets. ISG15, interferon-stimulated gene 15; OASL, 2’-5’-oligoadenylate synthetase; IRF3, Interferon regulatory factor 3; IRF7, Interferon regulatory factor 7; CXCL2, CXC motif chemokine ligand 2; APOA1, Apolipoprotein A1; APOC3, Apolipoprotein C3; FABP1, Fatty Acid Binding Protein-1; FABP2, Fatty acid binding protein 2; ACSL3, acyl-CoA synthetase long-chain family member 3; SLC27A2, Solute carrier family 27 member 2; ^a,b,c,^ Values within a column not sharing a common superscript letter indicate significant difference at *P* < 0.05.

PEDV infection in piglets usually induces the expression of inflammatory factors and the disturbance of lipid metabolism. To further investigate the effects of LGG powder supplementation on the immune and lipid metabolism status of PEDV-infected piglets, we performed qRT-PCR analysis for some anti-inflammatory and lipid metabolism-related genes. The regulation of jejunal intestinal gene expression is shown in **Figure 4**. Compared with none-challenged piglets, the PEDV infection significantly increased expression levels of PEDV M, PEDV N, OASL, CXCL2, IL-8 and IL-1β (*P*<0.05), and decreased expression levels of ISG15, IRF3, APOA1, APOC3, FABP1, FABP2, ACSL3 of SLC27A2 in the jejunum (*P*<0.05). LGG Powder supplementation decreased expression levels of PEDV M, PEDV N, OASL, CXCL2, IL-8 and IL-1β (*P*<0.05), and increased expression levels of ISG15, APOA1, FABP1, FABP2 (*P*<0.05).

### 3.4 Jejunum transcriptome

**Figure 5:**
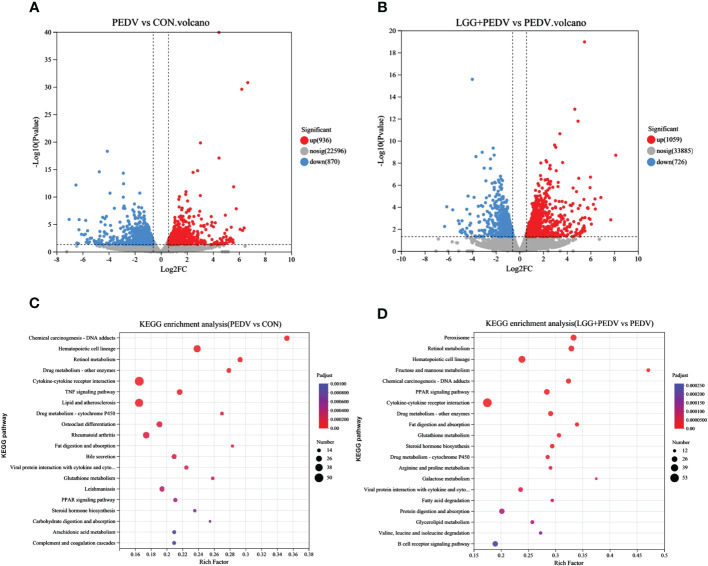
Jejunum mucosal transcriptome. **(A)** Volcano plot of DEG in PEDV and CON group. Up-regulation, none difference, and down-regulation are represented by red, grey, and blue dots, respectively. **(B)** Volcano plot of DEG in LGG+PEDV and PEDV group. **(C)** KEGG pathway enrichment analysis of DEG in PEDV and CON groups. The abscissa is the name of the pathway, and the ordinate is the *P*-adjust showing the enrichment of each pathway; the number of genes in the circles shows the number of differentially expressed genes enriched in the pathway. **(D)** KEGG pathway enrichment analysis of DEG in LGG+PEDV and PEDV group The abscissa is the name of the pathway, and the ordinate is the *P*-adjust showing the enrichment of each pathway; the number of genes in the circles shows the number of differentially expressed genes enriched in the pathway.

**Figure 6:**
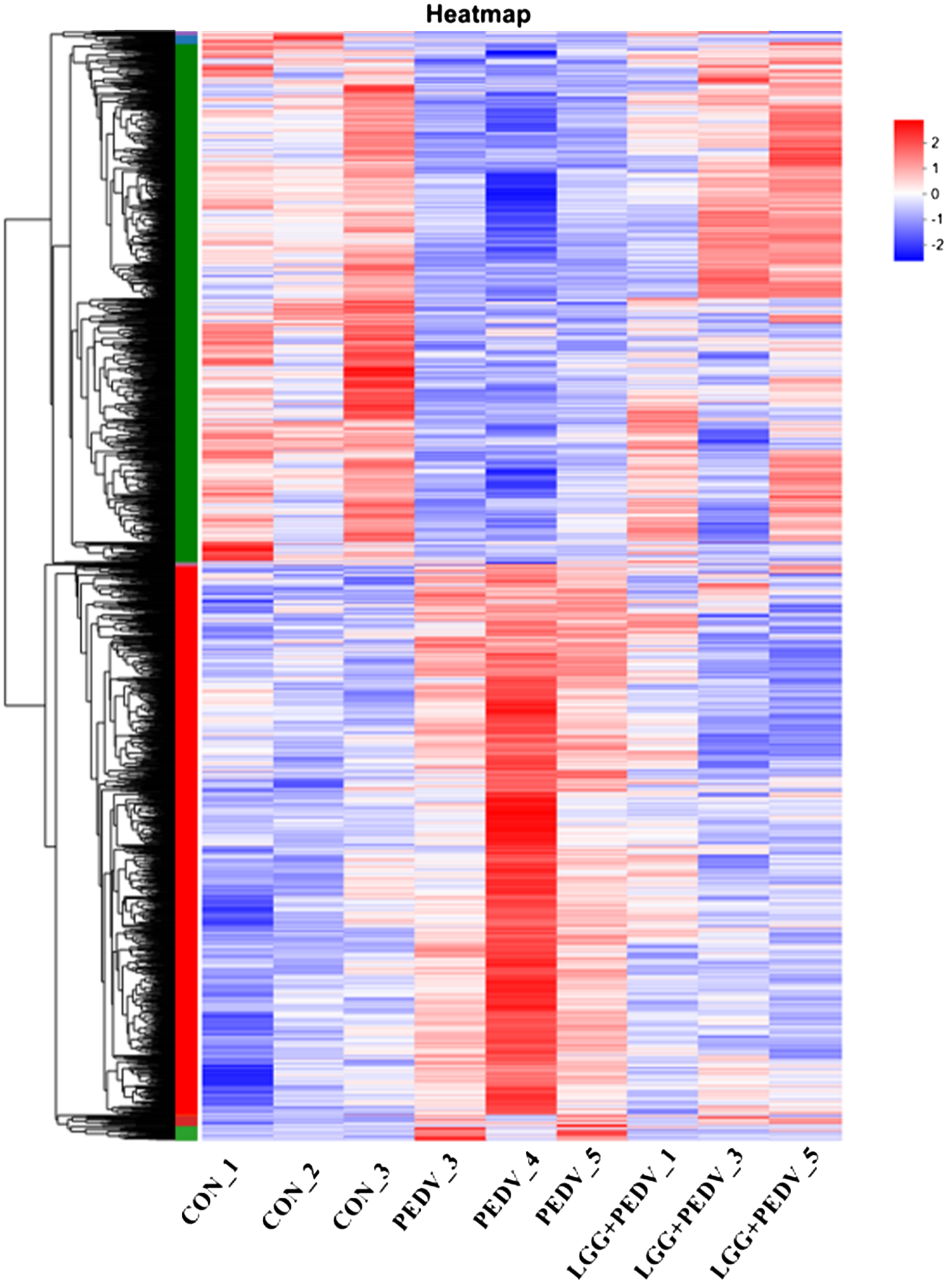
The red color indicates up-regulated genes, and the green color indicates genes showing down-regulated. Each row represents a gene, and each column is a sample.

Transcriptome sequencing technology (RNA-seq) is an effective tool for mining differentially expressed genes and regulatory networks in specific environments, thus we used transcriptome sequencing technology to explore the key pathways of LGG powder action on PEDV-infected piglets. Compared with none-challenged piglets, 1806 DEGs (936 up-regulated and 870 down-regulated) were identified in the PEDV group (**Figure 5A**). KEGG enrichment analysis showed the most significant pathway including Chemical carcinogenesis-DNA adducts, Hematopoietic cell lineage, Retinol metabolism, Drug metabolism-other enzymes, Cytokine-cytokine receptor interaction, TNF signaling pathway, Lipid and atherosclerosis, Drug metabolism-cytochrome P450, Osteoclast differentiation, Rheumatoid arthritis, Fat digestion and absorption, Bile secretion, PPAR signaling pathway, et al. (**Figure 5C**).Compared with the PEDV group, 1785 DEGs (1059 up-regulated and 726 down-regulated) were identified in the LGG+PEDV group (**Figure 5B**). KEGG enrichment analysis showed the most significant pathway including Peroxisome, Retinol metabolism, Hematopoietic cell lineage, Fructose and mannose metabolism, Chemical carcinogenesis-DNA adducts, PPAR signaling pathway, Cytokine-cytokine receptor interaction, Drug metabolism-other enzymes, Fat digestion and absorption, Glutathione metabolism, steroid hormone biosynthesis, Drug metabolism-cytochrome P450, Arginine and proline metabolism, Galactose metabolism, Fatty acid degradation, Protein digestion and absorption, Glycerolipid metabolism, et al. (**Figure 5D**). Clustering heatmap indicated that DEG samples selected from each group presented a similar expression pattern (**Figure 6**).

### 3.4 Verification of transcriptome sequencing results by qRT-PCR

**Figure 7:**
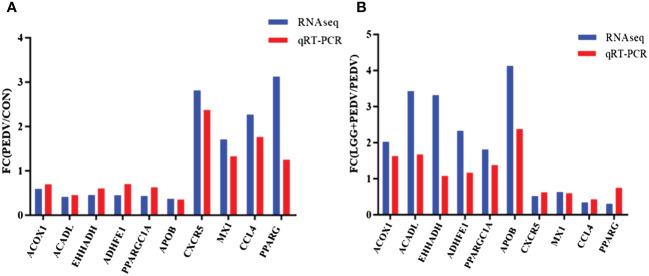
**(A)** Fold change indicates the ratio of average expression of PEDV-affected mRNA relative to controls. **(B)** Fold change indicates the ratio of average expression of LGG-affected mRNA relative to PEDV.

The transcriptome data and qRT-PCR results are shown in **Figure 7**. Ten genes identified by qRT-PCR were consistent with the transcriptome data, and the fold changes were basically the same, which indicated the consistency between the qRT-PCR and transcriptome data.

## 4 Discussion

The digestive and absorptive functions of piglets are closely related to the morphology integrity of the intestinal mucosal, and the villus height, crypt depth and villus height/crypt depth ratio are important indicators of intestinal morphology structure and integrity. The shortening of small intestinal mucosal epithelial villi and deepening of crypts represent shrinkage of intestinal mucosal epithelial villi and decreased absorption capacity; the increase in VH/CD represents an increase in the specific area of the mucosal and an increase in the digestive and absorptive capacity (Puppel et al., 2015; Choi et al., 2020). In addition, It has been shown that the addition of *Lactobacillus delbrueckii* to the diet significantly increased the ratio of villus height to crypt depth in the jejunum and ileum, significantly decreased crypt depth in the jejunum, and increased villus height in the ileum in weaned piglets (Wang et al., 2021). Dietary administration of 5 × 10^10^ CFU/kg *Lactobacillus Royale LR1* (LR1) to weaned piglet diets significantly increased villus height of the ileum as well as the ratio of ileal and jejunal villus height to crypt depth (Yi et al., 2018). A study by Wang et al. showed that the mRNA expression of intestinal occludin and ZO-1, and the protein expression level of occludin were increased by *L. plantarum* 299v treatment (Wang et al., 2019). *Lactobacillus* possibly improves intestinal barrier function by up-regulating the expression of Tight junction proteins, which are critical structural components of intestinal epithelial cells. The beneficial effect of *Lactobacillus rhamnosus* GG on improving the mucosal barrier function in the intestine of weaned piglets has been proved (Mao et al., 2016). Interestingly, In this experiment, supplementation of *Lactobacillus rhamnosus* GG powder significantly alleviated the intestinal morphology of PEDV-infected 7-day-old piglets, indicating that the *Lactobacillus rhamnosus* GG could play a function in the early life of piglets.

T-SOD and CAT belong to the main radical scavenging capacity of the organism, and their activity directly reflects the antioxidant capacity of the organism. MDA is a lipid peroxidation product, and its content directly indicates the degree of lipid peroxidation of the organism, which has cytotoxicity (Shi et al., 2014). MDA is an important index for the evaluation of lipid oxidation damage in the body, and can also indirectly reflect the damage of intestinal mucosal cells. MPO is a protein enriched in neutrophils, and higher activity indicates a more intense inflammatory response (Kolářová et al., 2021). In the present study, PEDV infection resulted in a significant decrease in CAT and T-SOD activities, a significant increase in MPO activity, and a significant increase in MDA content in the intestine, suggesting that PEDV successfully induced oxidative damage in the intestinal mucosa of piglets. Supplemented with *Lactobacillus rhamnosus* powder significantly increased CAT and T-SOD activity and reduced MDA content in the intestine. Consistently, previous studies have found that erythrocyte CAT and T-SOD activities were elevated in piglets fed *Lactobacillus* FT28, which effectively increased the antioxidant level of piglets (Dowarah et al., 2018). Dietary administration of *Lactobacillus rhamnosus* GG to the diet can reduce the damage of deoxynivalenol to the renal antioxidant system of piglets and effectively alleviate the oxidative damage of the kidneys (Ma et al., 2022). *Lactobacillus rhamnosus* GG can inhibit oxidative stress and apoptosis, thereby counteracting rotavirus-induced intestinal epithelial cell damage (Buccigrossi et al., 2022). In addition, supplementation of the diet with *Lactobacillus rhamnosus* GG (LGG) has been reported to significantly increase T-SOD activity and significantly decrease MDA levels in foal plasma (Shi et al., 2023). The results of this experiment were similar to those of previous studies, indicating that the addition of *Lactobacillus rhamnosus* GG powder to the diets could improve the antioxidant enzyme activities, enhance the body’s ability to scavenge free radicals, and alleviate the oxidative damage of the body in PEDV-infected piglets.

Transcriptomic techniques provide us with a powerful tool to discover potential biochemical pathways and reveal biological insights. The present study focused on the TNF signaling pathway, PPAR signaling pathway, fat digestion and absorption, and fatty acid degradation. The innate immune response plays an important role in the defense of mammalian cells against viral infections. After viral infection, an intrinsic antiviral immune response is initiated, and the body synthesizes interferon to induce the expression of antiviral molecules and to interfere with viral replication (Yang et al., 2022; Jung et al., 2023). In this study, PEDV infection significantly increased the content of Mx1 and OASL, whereas *L. rhamnosus* LGG supplementation powder decreased the levels of Mx1 and OSAL, similar to the control group. The recent study has shown that inflammatory cytokines, and small amounts of pro-inflammatory cytokines may have a protective effect against viral invasion (Huan et al., 2017), however, overproduction of cytokines disrupt the host immune response and affects the integrity of the small intestinal barrier, further exacerbating intestinal immune barrier damage (Liu et al., 2012), therefore, effective control of inflammatory cytokines expression is important for piglet gut health. The TNF signaling pathway may have been activated due to PEDV infection in this study, and this was also corroborated by the results of qRT-PCR. In the jejunal mucosa, we detected a significant increase in the mRNA expression of the inflammatory factors downstream of the TNF signaling pathway, IL-β, CXCL2, and IL-8, which suggests that a severe inflammatory response was induced in the cells of the jejunal mucosa (Qian et al., 2022). Whereas, *L. rhamnosus* GG supplementation decreased the levels of IL-β, CXCL2, and IL-8, suggesting its effects on alleviation of the inflammation. It has been reported that supplementation of piglets with lactic acid bacteria-fermented formula milk significantly decreased the levels of pro-inflammatory cytokines and inhibited the expression of inflammatory cytokines CXCL9 and CXCL10 in the colonic mucosa (Lin et al., 2023), and supplementation of ETEC-infected piglets with *L. rhamnosus* LB1 decreased the levels of TNF-α and IL-1β (Wu et al., 2021). *Lactobacillus rhamnosus* inhibits E. coli-induced expression of pro-inflammatory cytokines TNF-α and IL-1β in piglets (Li et al., 2012). Similar results were obtained in the present study, suggesting that *L. rhamnosus* GG powder may alleviate the inflammatory response of PEDV-infected piglets by down-regulating the expression of TNF signaling pathway.

Peroxisome proliferator-activated receptors (PPARα, PPARβ/δ, and PPARγ) belong to the transcription factor family, is a nuclear hormone receptor activated by fatty acids and their derivatives (Peng et al., 2021). PPARα plays a role in removing or recycling cellular lipids by regulating the expression of genes related to lipid metabolism in liver and skeletal muscle, PPARβ/δ are involved in cell proliferation and lipid oxidation, and PPARγ can promote adipocyte differentiation. In this study, PEDV infection significantly decreased the levels of mRNAs of key differentially expressed genes in the PPAR signaling pathway and the fat digestion and absorption pathway, such as ACADL, ACOX1, APOA1, APOC3, EHHADH, FABP1, FABP2, ACSL3, SLC27A2, APOB, etc, which was in line with the recent studies, that PEDV infection significantly suppressed the expression of genes related to lipid metabolism (Mao et al., 2022). Long-chain acyl-CoA dehydrogenase (ACADL) is the first step in fatty acid oxidation and plays a vital role in long-chain fatty acid β-oxidation, and its deficiency or deletion also causes mitochondrial metabolic disorder and affects lipid metabolism (Ji and Friedman, 2021). ACOX1 gene is strongly associated with fatty acid metabolism and triglyceride deposition in piglets (Zuo et al., 2007). Apolipoprotein A1 (APOA1) is the main apolipoprotein of plasma HDL and is involved in the regulation of intracellular lipid levels (Cochran et al., 2021), and it has also been reported that APOA1 is associated with the development of inflammatory and oxidative processes in the body (Madahian et al., 2014). Apolipoprotein A-IMilano (apoAIM), a naturally occurring cysteine mutant of apoAI with dimers as its effective form, and recombinant HDL-APOA1M (rHDL- APOA1M) formed from APOA1M and HDL has significant anti-inflammatory and anti-oxidant functions (Zhang et al., 2015). In this study, the mRNA expression of the APOA1 gene and other genes related to lipid metabolism was significantly down-regulated in the jejunal mucosal tissues of piglets infected with PEDV, suggesting that PEDV infection caused disorders of intestinal lipid metabolism and inflammatory responses in piglets. Supplementation of PEDV piglets with *Lactobacillus rhamnosus* GG powder significantly up-regulated the expression of mRNAs of key differentially expressed genes in the PPAR signaling pathway and the fat digestion and absorption pathway, such as ACADL, ACOX1, APOA1, APOC3, EHHADH, FABP1, FABP2, ACSL3, SLC27A2, APOB, etc., and then alleviated the disorders of lipid metabolism in the intestinal tract due to PEDV infection. The results were similar to a previous study conducted on Intestinal porcine cells (IPEC‐1) that *L. plantarum* inhibited the reduction in peroxisome proliferator‐activated receptor‐γ (PPAR‐γ) expression caused by ETEC K88 (Yang et al., 2021). Differently, Oral administration of *Lactobacillus plantarum* 299v down-regulated PPAR signaling pathway gene expression in the ileum of weaned male 4-week-old pigs (Hulst et al., 2015). Such variable results might be attributed to the variations in the types of probiotics, animals, cells, and experiment times.

## 5 Conclusion

Supplementation of *Lactobacillus rhamnosus* GG powder improved intestinal morphology, enhanced intestinal antioxidant capacity, and alleviated jejunal mucosal inflammation and lipid metabolism disorders in PEDV-infected piglets, which may be regulated by *Lactobacillus rhamnosus* GG powder by altering the expression of TNF signaling pathway, PPAR signaling pathway and fat digestion and absorption pathway.

## Data availability statement

The datasets presented in this study can be found in online repositories. The names of the repository and accession number(s) can be found below: NCBI; PRJNA1099660.

## Ethics statement

The animal studies were approved by The Institutional Animal Care and Use Committee of Wuhan Polytechnic University. The studies were conducted in accordance with the local legislation and institutional requirements. Written informed consent was obtained from the owners for the participation of their animals in this study.

## Author contributions

ZX: Writing – original draft. QZ: Formal analysis, Writing – review & editing. MW: Data curation, Writing – review & editing. YZ: Investigation, Writing – review & editing. ZL: Investigation, Writing – review & editing. HL: Project administration, Writing – review & editing. CY: Project administration, Writing – review & editing. XZ: Project administration, Writing – review & editing. DZ: Data curation, Writing – review & editing. LW: Data curation, Writing – review & editing. YH: Funding acquisition, Writing – review & editing. TW: Funding acquisition, Writing – review & editing.
